# Scrofulus Glands

**Published:** 1889-05-18

**Authors:** 


### SCROFULOUS GLANDS.

An important discussion took place a week or two ago, at the Harveian Society of London, on the subject of scrofulous glands. Many parents know by painful experience the distressing anxiety with which they see the gradual development of such glands—particularly in the neck—and the prolonged and sometimes unavailing efforts at cure made by the surgeon. The expressions of opinion at the Harveian Society were on the whole decidedly encouraging, The president, Dr. Thomas Buzzard, was strongly inclined to believe that scrofulous glands were not nearly so common in these times as formerly. That was a most consolatory statement. Of all methods of dealing with possible disease, prevention is surely the best. He had himself seen, he said, scrofulous glands actually cultivated at school by bad hygienic management. The main causes of the disease he believed to be two; and this all non-professional readers who have families should carefully note. The two causes were insufficient milk in the dietary, and defective ventilation. These two causes, in the case of ordinary people are perfectly removable; and, if it be true, as Dr. Thomas Buzzard, a very high authority, believes, that they are the *main* causes, then scrofulous glands should to a large extent disappear from among the prosperous classes. It is not difficult for such persons, even in large towns, to procure an abundant supply of good milk. The fresh air question is less easy; but even in regard to fresh air it is wonderful what may be done by constant and intelligent watchfulness on the part of the mother or other responsible person. The treatment recommended by some of the best authorities was the prompt cutting out of the glands. Painting with iodine was declared by Mr. Christopher Heath to be in many cases entirely useless. He had no faith in cauterisation. Partial or complete removal was held by most to be the best method of treatment when the disease was fairly established. But in the early stages, milk, cod-liver oil, fresh air, and the local application of Kreuznach water it was agreed were highly serviceable. The discussion was of value to outsiders as showing the folly of waiting to see how a particular case will go on before seeking medical aid. At the very first sign of glandular enlargement competent advice is to be sought at once. The neglect of such a course may result in permanent enfeeblement of the health and lifelong disfigurement.

